# Neuraxial biomechanics, fluid dynamics, and myodural regulation: rethinking management of hypermobility and CNS disorders

**DOI:** 10.3389/fneur.2024.1479545

**Published:** 2024-12-10

**Authors:** Nicole Frost, S. Jade Barclay

**Affiliations:** ^1^Flex-Ability Physio, Wollongong, NSW, Australia; ^2^Connected Health Alliance, Wollongong, NSW, Australia; ^3^Neuromuscular Imaging Research Lab, The Kolling Institute, North Sydney Local Health District, St Leonards, NSW, Australia; ^4^Hypermobility and Performance Lab, Faculty of Medicine and Health, The University of Sydney, Sydney, NSW, Australia

**Keywords:** hypermobility, multidisciplinary, multimorbidity, biomechanics, neurology, neurosurgery, nervous system, cerebrospinal fluid

## Abstract

Individuals with joint hypermobility and the Ehlers-Danlos Syndromes (EDS) are disproportionately affected by neuraxial dysfunction and Central Nervous System (CNS) disorders: such as Spontaneous Intracranial Hypotension (SIH) due to spinal cerebrospinal fluid (CSF) leaks, Upper Cervical Instability (UCI; including craniocervical or atlantoaxial instability (CCI/AAI)), Occult Tethered Cord Syndrome (TCS), Chiari Malformation (CM) and Idiopathic Intracranial Hypertension (IIH). The neuraxis comprises the parts of the nervous system (brain, nerves, spinal cord) along the craniospinal axis of the body. Neuraxial tissue includes all tissue structures that comprise, support, sheath, and connect along the neuraxis and peripheral nerves. Altered mechanical loading or vascular supply of neural structures can adversely impact neural health and conductivity, with local and remote effects on inflammation, venous congestion, and muscle control. With EDS characterized by altered structure of the connective tissues found throughout the body including the neural system, altered mechanical properties of the central nervous system (CNS) and its surrounding tissue structures are important considerations in the development and diagnostics of these CNS disorders, as well as response to therapeutic interventions. Experts have identified a need for neuraxial curriculum in medical education and hypermobility-adapted treatment approaches in pain management, neurosurgery, anesthesiology, hematology, gastrointestinal surgery, dermatology, cardiology, dentistry, gastroenterology, allergy/immunology, physical therapy, primary care, radiology and emergency medicine. This paper reviews the interactions between neuraxial biomechanics and pathology related to CNS disorders seen commonly with EDS. First, we provide a concise synthesis of the literature on neuraxial kinematics and fluid dynamics. We then discuss the interplay of these biomechanics and their involvement in clinically-relevant diagnoses and overlapping symptom presentations, modeling physiological reasoning to highlight knowledge gaps, support clinical decision-making, improve multidisciplinary management of hypermobility-associated complexity, and add weight to the call for medical education reform.

## 1 Introduction

Neurological conditions recently surpassed cardiovascular and musculoskeletal conditions to become the leading disease burden globally, affecting 1 in 3 people in 2021 (1). Hypermobile patients are disproportionately affected by neuraxial disorders (2–4)—including Spontaneous Intracranial Hypotension due to spinal cerebrospinal fluid leaks, Upper Cervical Instability, Occult Tethered Cord Syndrome, Chiari Malformation and Idiopathic Intracranial Hypertension (2, 3)—with interacting pathology, dysregulation, and complexity that transcends conventional silos in healthcare. Rethinking how various changes in neural, fascial and vascular tissue biomechanics interact, the neuraxial-myodural complex provides a framework that can clarify the mechanisms at play behind symptom cascades and unexpected treatment responses. The dynamics of the neuraxial-myodural complex—the combined interactions of neuraxial tissue and the myodural bridge—in each hypermobile patient present an additional challenge to diagnostic assessment and treatment planning as the extracellular matrix and tissue components may deviate from expected physiology while also offering opportunities for preventative and therapeutic intervention. Neurological function requires the neural system to accommodate changes in mechanical loading while maintaining allostasis of metabolic, fluid, and conductive functions, dependent on the capacity and integrity of neural and vascular connective tissue. Altered connective tissue structure, volume, and production impacts neurological function and neuraxial biomechanics, which can be adaptive or maladaptive. Small changes in fluid dynamics (including tissue osmolarity and fluid flow, volume and pressure) or kinematics (motion of body parts) can have widespread and long-term effects. Compromised neuraxial biomechanics in tissue or fluid components at any point in the system can impact the integrity of mechanical tissue properties, and subsequent capacity for neural conduction and CNS response to everyday changes in posture, body motion, or physiological function. When overlooked or maladaptive, this interplay contributes to cascades of “unrelated” pathology, ineffective treatments, and compounding stigma. Understanding the associations between mechanical and functional interactions—between spinal joint function and sensorimotor control, the myodural bridge, dural and cord motion, dural compliance, CSF and vascular pressure—is essential to improve diagnostic decisions, treatment plans, and therapeutic outcomes, and to avoid preventable harm to patients with altered connective tissue.

Biomechanical differences predispose people with hypermobility to higher risk of neuraxial comorbidities (often multiple simultaneously) and neurological dysfunction (both central and peripheral) (2, 5). With increased rates of cervical spine instability and related complications in hypermobile patients, experts have identified a need for neuraxial curriculum (6) (and related reform) in medical education (7) and hypermobility-adapted treatment approaches (8): in pain management (9), neurosurgery (7), anesthesiology (6), hematology (10), gastrointestinal surgery (11), dermatology (12), cardiology (13), dentistry (14), gastroenterology (15), allergy/immunology (16), physical therapy (17), primary care (18, 19), radiology and emergency medicine (20).

Neuraxial tissue must accommodate greater range of spinal motion and spinal canal elongation in patients with hypermobility (21). Hypermobility increases the risk of injury and systemic impairments in response to normal body movement, with significant biomechanical, neuromuscular, and neurovascular vulnerabilities in the neuraxis and associated structures. Increased demand in response to hypermobile patterns of movement and mechanical load, when combined with variations in tissue compliance and fragility, likely compromises neural compliance, nutrition, metabolic waste clearance, or elasticity limits of the impaired and overloaded neuraxial structures.

Structures in and around the craniocervical junction are particularly vulnerable with hypermobile necks and spines. Spinal hypermobility (increased physiological joint range of motion due to anatomical and tissue variance) or mechanical instability (excessive accessory joint motion due to reduced capacity of tissue to stablize or hold a joint in place) can have serious consequences with higher segments having higher consequences. Finite segment analysis shows that hypermobility of upper cervical segments increases mechanical loading of the spinal cord (22). Increased cord stress is associated with higher ranges of atlanto-axial rotation and lateral translation, and higher rotation is associated with compromised cranial vascular supply and vertebral artery patency (23, 24). Perioperative planning and diagnostic investigations should consider the potential for articular subluxation or hemorrhagic complications during routine patient transfers, cumulative exposure to radiology contrast, anesthesia resistance, delayed wound healing, and reduced kinematic control when assessing or treating conditions associated with connective tissue or cervical musculoskeletal dysfunction along with associated conditions such as dysautonomia, whiplash, migraine, tinnitus, and other (6, 10, 11, 25, 26).

This paper reviews the interactions between neuraxial biomechanics and pathology related to CNS disorders seen commonly with hypermobility and related conditions including the Ehlers-Danlos Syndromes (EDS). First, we provide a concise synthesis of the literature on neuraxial kinematics and fluid dynamics. We then discuss the interplay of these biomechanics and their involvement in clinically-relevant diagnoses and overlapping symptom presentations, modeling physiological reasoning to highlight knowledge gaps and equip clinicians to manage this complexity in practice. Enriched understanding of neuraxial biomechanics and fluid dynamics in EDS serves to improve long-term health outcomes, support clinical decision-making, avoid preventable harm, and guide complex care planning. By clarifying the functions, consequences, and interplay of neuraxial-myodural dynamics—kinematic, proprioceptive, fluid, and tissue changes—we aim to support clinical decision-making, improve multidisciplinary management of hypermobility-associated complexity, and add weight to the call for medical education reform.

## 2 Neuraxial kinematics and biomechanics

The neuraxis is the axis of the central nervous system (CNS), comprising the brain and spinal cord, and continuous mechanical tract of supporting tissue structures anchored at many points including the craniocervical junction and coccyx. The neural system is academically divided into central and peripheral neural systems (PNS) while in fact being connected structurally and by the flow of fluids, including CSF, throughout both CNS and PNS (27). Clinically, injury, impairment, and instability may present differently in each of the major neuraxial transition junctions—atlantoaxial to cervical, cervical to thoracic, thoracic to lumbar, lumbar to sacroiliac—and interact via this continuous tissue tract. As a mechanically continuous structure, a change in kinematics of any part of the neural tissue tract has the potential to change the forces operating on any other part of the tract. For full, asymptomatic function, all tissues of the neural tract must be able to accommodate the mechanical loads associated with bodily movement, postural changes relative to gravity, and fluid dynamics of the CSF and vasculature, while maintaining the capacity for impulse conduction. Changes in mechanics anywhere along the tract may therefore contribute to pathological neural function in both local and remote areas of the neural system (28).

### 2.1 Neuraxial length adaptation

The neuraxis can be deformed during head and spinal movement, with neurons and associated vasculature and connective tissues all potentially undergoing mechanical deformations. Spinal motion involves changes in the length of the central canal to which the neuraxis must adapt. In an average human spinal column, the central canal is estimated to be 5–9 cm longer in trunk flexion than extension, with a greater length change posteriorly than anteriorly (29, 30). Both the anterior and posterior neuraxis lengthen in flexion and shorten in extension as the entire neuraxis is positioned to the same aspect of the axis of motion. The neuraxis shows less change in length than the bony spinal canal during flexion and extension, with a relative displacement of the end of the dural sac suggesting some degree of stretch (31). Nerve roots generally follow relaxed, curved paths in an extended spine, being drawn into straightened paths during flexion (31). During lateral flexion of the spine, the convex side of the canal will lengthen, and the concave shorten. During rotation of the spine, there is potential for spinal structures to encroach on the dural sac, with the canal narrowing at the craniocervical junction (due to atlas movement) and the cord narrowing at the thoracic level. Nerve roots also stretch up to 1 cm during rotation of adjacent vertebrae (31, 32).

Shortening of the spinal column involves telescoping and folding of tissues along the length of the neuraxis, while lengthening is normally accommodated via unfolding then elastic lengthening of the neural tract structures (30). The axons, blood vessels, and connective tissue fibers of the neuraxis, surrounding the neural cells and investing into the cord surrounding vascular structures, are arranged in various geometric networks that are able to accommodate length changes through shifts in the geometry of the fiber network, such as coiling or concertina like folds on shortening, with a sudden arrest of connective tissue length change occurring when the fiber networks are fully folded or opened (28, 30), thereby protecting the neural cells from excessive distortion. Dural tissue demonstrates greater resistance to longitudinal stretch than transverse as a consequence of the primarily cranio-caudal orientation of the collagen fibers, with variable stiffness in different regions of the spine and viscoelastic properties attributed to the presence of elastin fibers and ground substance of the extracellular matrix in addition to the collagen fibers (33, 34).

As a consequence of the mechanical continuity of the neural tract, movements of any part of the body, not only the spine, may influence the forces on the neuraxis remotely. Transmission of tension to the lumbar cord during cervical flexion is shown in cadaver studies, and animal studies demonstrate potential for distal limb movements to change tension in remote neuraxial tissues (29, 35, 36). A differentiated response of the dura to pathological forces has been described dependent on the pre-tensioning of the neuraxis, with a cranial or caudal force producing significant deformation of the dura over a small range when applied to slack dura (such as in spinal extension), while a pre-stretched dura required larger forces to produce deformation which transmits over greater distances (28).

### 2.2 Neuraxial tissue interfaces

**Figure 1:**
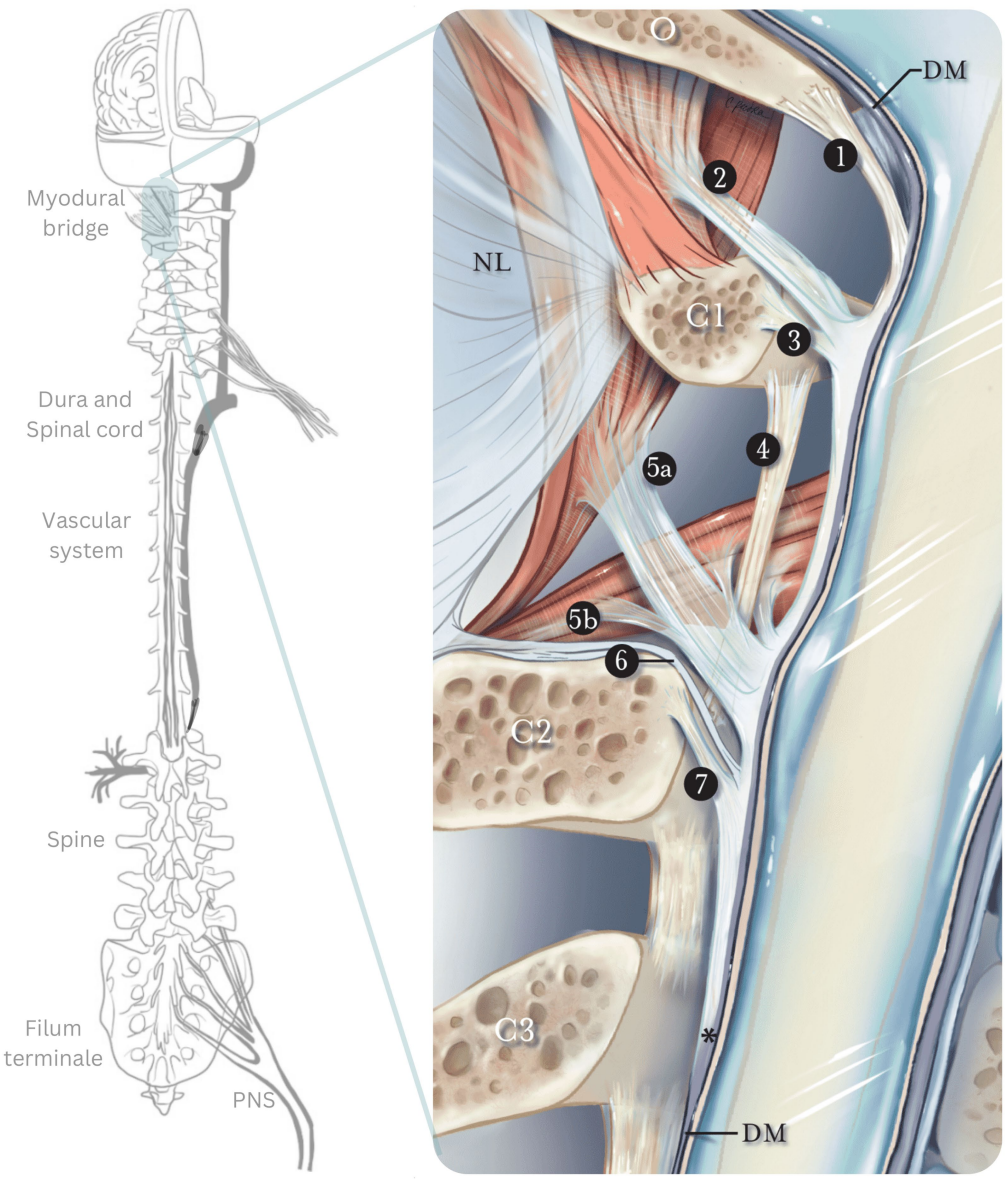
The myodural bridge. Sagittal section of the myodural bridge and its connections to the dura and spine. The posterior atlantooccipital membrane (1) extends from the occiput and coalesces with the dura mater at the cerebrospinal junction. The superior myodural bridge (2) merges with the superior vertebrodural ligament (3) of the atlas and fuses with the PAOM at the level of the atlantooccipital interspace. The inferior myodural bridge comprised of the rectus capitis posterior major fascia (5a) and obliquus capitis inferior fascia (5b) courses between the atlantoaxial ligamentum flavum (4) as bundles of dense fibers. The inferior myodural bridge fuses with the PAOM. The nuchal bridge (6) merges with the inferior vertebrodural bridge (7) and attaches to the PAOM. The PAOM terminates at the level of C3 after this transition point (*). The dura mater (DM) continues as an independent structure after that. O, occiput; C1, atlas; C2, axis; C3, third cervical vertebra; NL, nuchal ligament; PAOM, posterior atlantooccipital membrane. Adapted from Scali et al. (160).

During spinal motion, in addition to length change, there may also be relative motion between different tissue layers, such as the neuronal tissue, dural membranes, and spinal structures to a non-uniform degree throughout the spinal canal (37, 38). There are multiple locations where the relative motion between layers is restricted by direct attachments. The cranial dura is adhered to the inner surface of the cranial bones to varying degrees and is structurally continuous with the spinal dura. As depicted in Figure 1, the spinal dura is tethered to the anterior and lateral aspects of the spinal canal to varying degrees along its length by Hoffman’s ligaments and dorsally to the ligamentum flavum by a central septum, along with fixation to the margins of the intervertebral foraminae, and the nerve root complexes thereby mechanically attaching the neuraxis to the peripheral nervous system (31, 39). Within the dural sac, the cord is connected by a series of denticulate ligaments connecting the pia and dura mater which have been suggested to prevent excessive cord elongation based on animal studies (40). Additionally, an intermediate layer between the pia and arachnoid mater has been described which is most prominent dorsally, forming a series of septa within the spinal subarachnoid space (41). The intermediate layer is described as highly fenestrated in the lateral cord aspects and suggested to potentially influence movement between the cord and arachnoid mater as well as potentially dampening pressure fluctuations. Anatomy diagrams tend to oversimplify this; it’s complex and overcrowded in the craniocervical junction and the epidural space.

**Figure 2:**
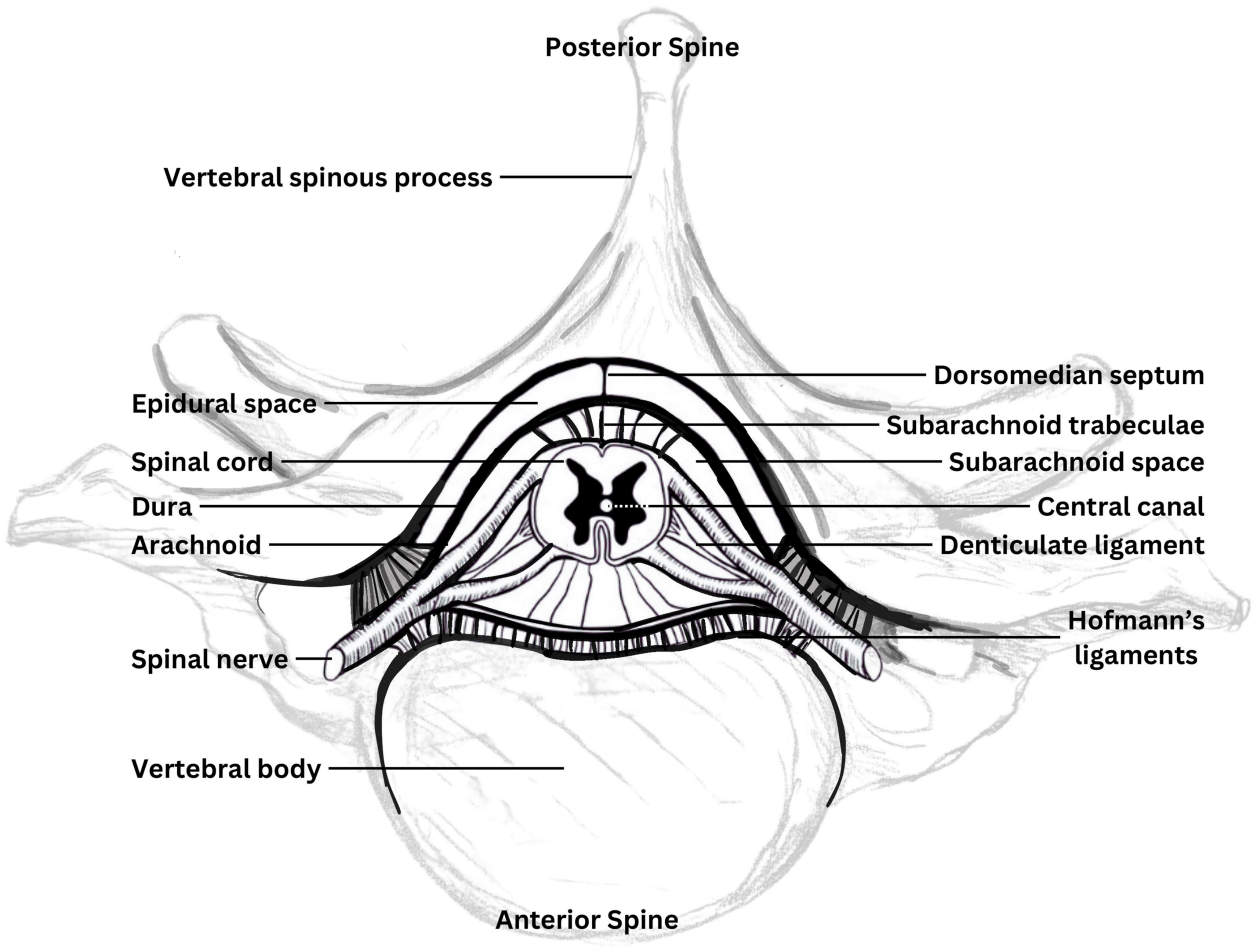
Components of the neuraxis. Diagrammatic cross-section of the spinal canal contents and immediately surrounding bony elements, showing attachments of the neuraxis. Adapted from Butler (35).

At the craniocervical junction, the spinal dura mater attaches to the skull base, upper cervical vertebrae, and suboccipital musculature (rectus capitis posterior major, rectus capitis posterior minor and obliquus capitis inferior), by a complex of membranous attachments passing through the occipito-axial and atlanto-axial interspaces; this complex is the myodural bridge, depicted in Figure 2 (42). The action of the suboccipital muscles connected to the dura via the myodural bridge may influence CSF pressure and flow and control the folding of the dural membranes during spinal motion (43, 44), Caudally, the meningeal tissues and cord are anchored via the fibrovascular filum terminale, a structure usually demonstrating greater elasticity than the cord, extending from the conus medullaris to the dorsal aspect of the coccyx (38). Through these direct connections between the neuraxial tissue and spine, the neuraxial motion and vertebral motion can directly influence each other and both influence the myodural bridge and filum terminale (42, 45, 46).

The volume of the epidural space varies at different levels of the spine with the cord noted to occupy less than half of the canal volume at the C1 level, compared to around three quarters at C6 (47). Surrounding the dura, in the spinal epidural space, is the internal venous plexus embedded in epidural fat deposits (48). The spinal epidural plexus, which occupies much of the epidural space (31) is described as a series of longitudinal plexuses with interconnecting venous rings at each vertebral body level, providing drainage for the cord and vertebral bodies. It has been suggested that the compressibility of the epidural plexus could offer the cord some protection against other structures encroaching into the canal during movement (such as bulging spinal ligaments) and buffering of changes in canal pressure during motion (31).

The tissues which may interface in direct contact with the CNS in the spinal canal, and therefore influence neuraxis motion, vary as the cord moves within the canal under the influence of both joint motion and gravity. In horizontal body alignments, the untensioned neuraxis (such as in a neutral or extended spine posture), will drop onto the lower surface of the spinal canal under the influence of gravity: the posterior aspect of the canal in supine, or the lowermost side of the canal in lateral lying. By contrast, in the flexed spine, when the neuraxis is under tension, it will be lifted away from the lower canal surface. Thus, combinations of body posture relative to gravity and segmental alignment of the spinal and limb musculoskeletal system can jointly influence the degree of contact between the neuraxis and surrounding structures, both anatomical and pathological (28). Factors which alter the relative displacement of tissue layers, such as epidural scarring, may alter the relative motion between tissues and the transmission and dissipation of applied forces (30).

### 2.3 CNS fluid biodynamics

The neuraxis is influenced by fluid dynamics of cerebrospinal fluid, lymphatic, glymphatic, and vascular systems; fluid pulsations, volume, pressure, directional forces, osmolarity, interacting hydrodynamics and fluctuating permeability of surrounding tissue (7). Due to the relative rigidity of the cranium and spinal canal and incompressibility of their neural contents, the dural sac with its contained CSF and the venous systems display interrelated dynamics responding to changes in each other’s volumes (described by the Monroe-Kellie hypothesis) (49), as well as to changes in intracranial, intrathoracic and intra-abdominal pressures (50). The buoyancy effect of the CSF normally reduces the downward displacement of the CNS under the influence of gravity in upright postures, significantly reducing the effective weight of the brain and likely also providing a hydraulic cushioning effect for the spinal cord (29, 40).

The understanding of CSF dynamics continues to evolve. According to modern hypotheses, CSF pulsates and circulates between the cranial ventricles and spinal cavities at the foramen magnum. CSF secretion and absorption occurs throughout the entire subarachnoid space, with regions of hyperosmolarity found throughout the CNS, cranial ventricles, and periventricular white matter (7, 50). No specific sites of active or passive absorption or secretion have been identified experimentally; contrary to the century-old classic hypothesis, the choroid plexus acts as neither pump nor primary source of CSF secretions. Instead, rapid changes in osmolarity has been identified as the main contributor to CSF dynamics in typical presentations (as osmolarity increases with inflammation). Impairments in osmolarity cause dural inflammation and craniospinal compensatory mechanisms (7). Constant fluid exchange and CSF turnover occurs in cerebral capillaries, and some absorption occurs through glial or glymphatic and perineural pathways following cranial nerves, passing to the cervical lymphatic system, and via the canalicular system from the subarachnoid space to the subclavian veins, interacting with other CNS hydrodynamics (51). Experimental findings also challenge the classic hypothesis of unidirectional flow of CSF in a craniocaudal direction, demonstrating bidirectional pulsating CSF movement influenced by body posture and vascular dynamics (7, 51, 52). The CSF continuously pulsates with respiratory and cardiac cycles with the brain and cord mechanically impacted by the oscillating pressure of these pulsations. The upper cord demonstrates cranio-caudal and anterior–posterior oscillations in a resting subject (45, 53, 54).

The pressure dynamics of the CSF will be a function of its volume (impacted by the rate of ingress and egress) as well as the compliance of the intradural space, both cranial and spinal as communicating compartments with differing properties, and influenced by vascular volumes. The relative compliance of the cranial compartment may differ from the spinal compartment, which is less constrained by a surrounding bony structure (55).

During a Valsalva maneuver (forced expiration against a closed glottis), the lower spinal dura rapidly narrows, displacing CSF upwards and distending the cervical dural sac, with an associated transient rise in intracranial pressure (50). Bilateral compression of the jugular veins, thereby restricting cranial venous outflow, has been associated with distension of the lower dural sac extending into sacral nerve roots suggesting caudal displacement of CSF (50, 52).

The mostly valveless spinal epidural venous complex allows bidirectional flow and can effectively connect the venous drainage from the cranium and lower body depending on pressure gradients (48). Cranial venous outflow is primarily via the internal jugular veins in the supine postures, transferring to primarily via the vertebral venous system in upright as the internal jugular veins collapse (56–59). Human cadaver and animal studies by Batson (60) demonstrated venous flow from the pelvic and chest regions, through the spinal system and into the cranium which was more pronounced under conditions of increased intra-abdominal pressure.

## 3 Interplay of neuraxial biomechanics in hypermobility

### 3.1 Kinematic interactions

**Figure 3:**
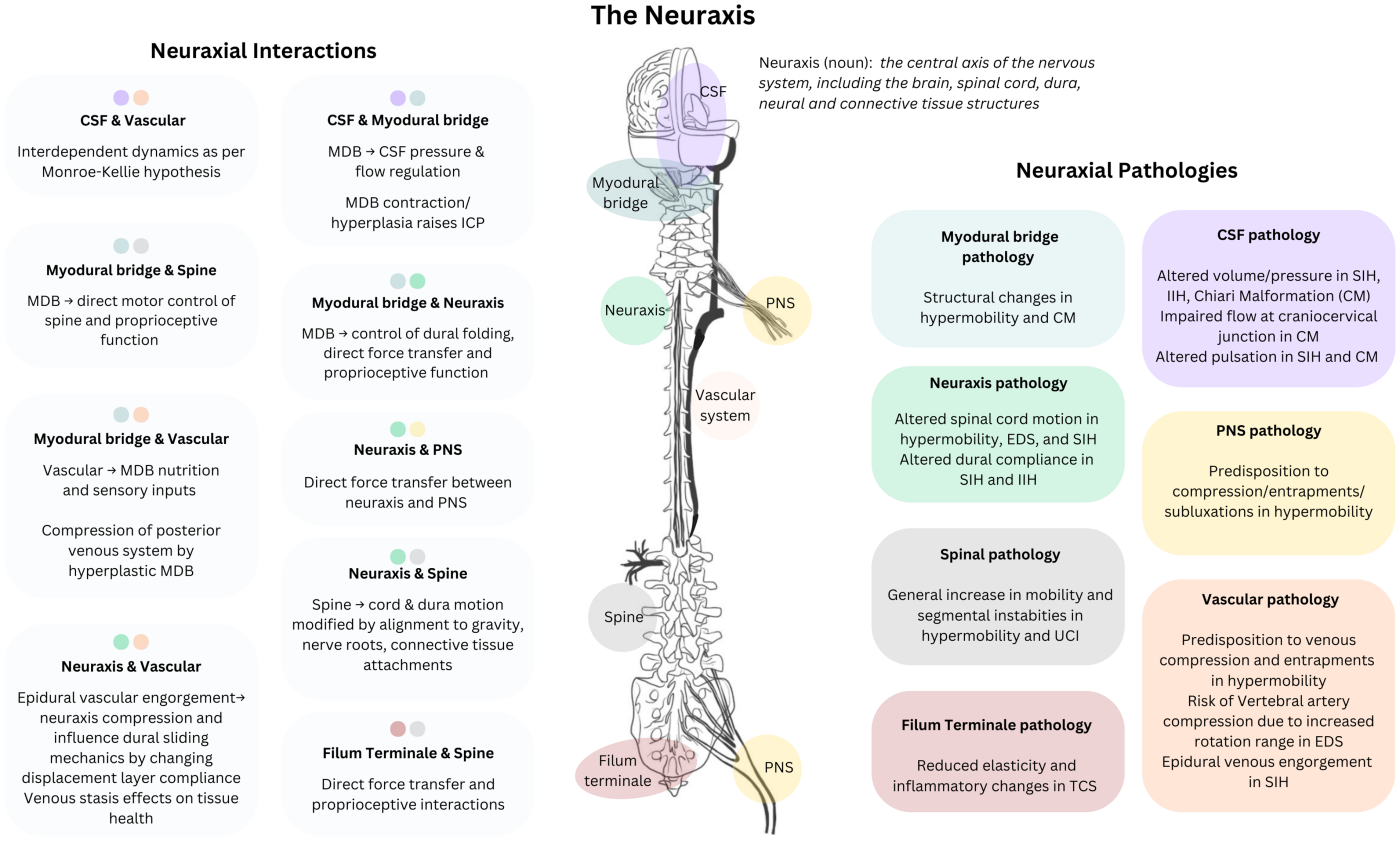
Neuraxial-myodural complex: Neuraxial components, pathologies, and interactions. Conceptual representation of the functional connections between elements of the neurological system and associated systems and pathological changes in hypermobility related disorders. CSF, cerebrospinal fluid; MDB, myodural bridge; TCS, tethered cord syndrome; SIH, spontaneous intracranial hypotension; EDS, Ehlers-Danlos Syndrome; IIH, idiopathic intracranial hypertension; CM, Chiari malformation; CNS, central nervous system; UCI, upper cervical instability; ICP, intracranial pressure.

Due to connective tissue differences in hypermobile individuals, CNS and fascial structures respond differently to increased demands in mechanical loading and the elastic, tensile and compressive forces associated with normal movement; increased stress on these structures is associated with reduced capacity to maintain vascular and perivascular patency under load (Figure 3) (23, 24). In patients with EDS and TCS, the filum terminale shows abnormalities in elasticity, collagen fibrils, and inflammatory cell invasion, and the myodural bridge shows swelling, disruption and altered orientation of collagen fibrils (61, 62). Collagen and inflammatory alterations, hyaluronan dysregulation, and impaired wound healing reduce capacity to recover structural integrity and mechanical function after damage; crucial peri-operative and post-operative considerations. Greater range of physiological motion and segmental spinal instabilities increase the risk of damage, and impaired or delayed wound healing reduces structural and mechanical integrity, further compounding instability and risk. This vicious cycle explains why an initial insult (injury or surgery) with hypermobile patients so often leads to recurring injuries or surgeries from which the patient does not quite recover as expected. Restoring kinematic control is needed early in both conservative injury rehabilitation and surgical intervention to address pressure differentials, reduce risks of secondary complications, and strengthen the capacity to recover structural integrity before adding surgical trauma to injury. Minimally invasive, strategic, coordinated treatment planning is essential with these conditions; the right treatments in the wrong order can have serious long term implications.

The oscillating motion of the spinal cord, in a cranio-caudal direction, results from the pulsations of CSF and blood driven by the effects of the cardiac and respiratory cycles (45, 58). At normal CSF volumes, CSF pressure may dampen spinal cord motion; reduced CSF volume in the spinal compartment may reduce this dampening effect (63). Cranio-caudal motion is altered in the presence of CSF leaks and cervical myelopathy. Increased cranio-caudal motion has been flagged as an early indicator of cervical myelopathy, “the cardinal pathophysiological change” before structural cord changes are apparent on MRI (45, 64). In CSF leaks, altered cardiac-synchronous pulsatile motion of the upper spinal cord structures and altered CSF flow may contribute to SIH symptoms by putting “increased mechanical strain on neural tissue and adherent structures” (63). Increased cardiac-synchronous pulsatility of the cervical cord has also been noted in patients with EDS (without CSF leak), together with an altered pattern of antero-posterior cord displacement within the canal during cervical spine movement (61). In the same study, electron microscopy identified structural changes in the myodural bridge, including fibril swelling and loss of integrity in fiber arrangements. Altered pulsatility and antero-posterior motion of the upper cord is posited to be “caused by laxity of [myodural bridges]s, as well as laxity of all other ligaments of the [cranio-cervical junction], including other dural suspension ligaments, [cranio-cervical junction] joint ligaments, and even the dentate ligaments” (61). Given the role of multiple connective tissue structures—stabilizing cranio-caudal motion and buffering pressure changes—tissue laxity throughout the neuraxis may reduce the resistance of the spinal cord to pressure fluctuations in EDS. Given the risks of surgery, anesthesia, and imaging contrast associated with altered connective tissue (25, 65, 66), assessment of changes in spinal cord motion may enable early detection of these conditions, providing a lower risk diagnostic option. Pathological spinal cord motion assessment is an emerging field, with future research needed into diagnostic and therapeutic applications: in diagnostics and early detection, to distinguish patterns with and without EDS, POTS, CNS pathology, CSF pressure disorders, spinal instabilities and other structural changes (such as Chiari Malformation, tethered cord, CCI/AAI); and in therapeutics: evaluate and compare the efficacy of surgical, kinematic, and other interventions by monitoring cord pulsatility and cord mechanics before and after treatment (or as a routine vital sign to aid early detection of multiple conditions, such as diabetes and cardiovascular disease).

### 3.2 Proprioception and motor control

Proprioception influences motor control and is targeted in conservative treatment for neuromusculoskeletal conditions (67, 68). The myodural bridge and filum terminale anchor the neuraxis and are associated with structural, proprioceptive, and functional changes throughout the whole body (46). Electrical stimulation of the filum terminale activates paraspinal musculature (46). In the legs, a diseased filum is associated with altered EMG responses in lower limb muscles. In the brain and spine, irritated meninges may cause contractions in suboccipital musculature (69). Impairment of these sensory functions can contribute to impaired muscular control of spinal and cranial articulations, dural folding, CSF dynamics, and damping of the mechanical loading of the spinal cord (43–46).

Animal models suggest myodural influences on intracranial CSF pressure, with increases in intracranial pressure during neck motion and contraction of obliquus capitis inferior (43, 44), and experimentally induced hyperplasia of rectus capitis posterior major and minor (39, 43). Co-occurring upper cervical instability with Chiari Malformation morphology is posited to increase activity of myodural bridge musculature, reducing dural compliance and thereby impacting the CSF pressure dynamics at the foramen magnum (70). This mechanism can lead benign Chiari Malformation to become symptomatic (42–44, 70), and may explain failure of decompression surgery to relieve symptoms in some patients (44, 71).

A bi-directional interplay of the myodural bridge function and CSF dynamics in the cranio-cervical region offers an explanation for suboccipital motor and sensory dysfunction in the presence of altered CSF dynamics. The dura is easily irritated by structural damage, mechanical overload, low CSF volume, extradural CSF, or local or systemic inflammation. Inflammation has also been found to increase permeability of mucosal and neuraxial tissue, including the dura and blood brain barrier (25, 42, 72); dura permeability necessarily impacts the CSF fluid within. Altered CSF volume or dynamics (IIH, SIH, Chiari) or irritation of the dura may alter myodural loading and function. Compounded by sensory, tissue, and proprioceptive sensitivity to changing fluid dynamics, altered myodural loading may subsequently impair proprioception, and muscle control, and contribute to further changes in dural irritation, dural tissue compliance, pressure dynamics, and risk of future leaks. This cluster of symptoms indicates the need for investigation of upstream factors as potential markers for early detection of CSF and neuraxial pathology, and potential intervention targets to address myodural or filum terminale impairment.

The myodural bridge transfers force from the suboccipital musculature to the cervical dura, with bi-directional compensatory effects. This can amplify and transfer impairment effects along the path of force transmission; reducing capacity to regulate muscle responses, resulting in over- or under-contraction relative to demand. Thus, dural irritation may contribute to (or exacerbate) impairments via the path of force transmission, including muscular regulation, sensory sensitivity, proprioceptive capacity, or myodural bridge function. Primary pathology of CSF pressure or flow may also drive changes in spinal mechanics via myodural force transmission through the suspensory structures—from the myodural bridge and filum terminale to the skeletal elements—or changes in sensory input and spinal motor control. Altered suboccipital muscle loading can transfer force via the myodural bridge to pathologically load the cervical dura; upper cervical laxity provides insufficient passive restraint of joints (even under normal load), increasing compensatory load on suboccipital musculature. Further research is needed to understand the role of the myodural bridge in regulating CSF and venous flow at the craniocervical junction, to understand associations between altered suboccipital muscle function and neuraxial tissues, bi-directional interactions between dural tissue and SIH, and to evaluate relevant diagnostics and therapeutic interventions.

Spinal stabilization deficits can directly load dura, influencing vascular, lymphatic and CSF circulation and pressures. Complex interactions between upper cervical function (neuraxial motion, joint stability, muscle regulation, force transmission) and CSF dynamics, modulated by myodural bridge function, offer insights into effective management strategies for CNS dysfunction and craniocervical structures in EDS. Multidirectional compromises of musculoskeletal, sensory, CSF dynamics and neuraxial motion provides an explanatory framework for clinical reasoning and rich targets for therapeutic and preventative strategies. Assessment and treatment of any one condition in this area must consider all elements and interacting functions of the neuraxial-myodural complex. Secondary deficits of proprioception and sensorimotor function of the upper cervical spine should be explored as exacerbating factors in primary CSF pressure disorders. Treatments to improve proprioception and kinematic motor control should be explored as a peri- and post-operative therapeutic target to improve overall functional recovery from surgery and leak repair, and reduce post-repair rates of CSF leak recurrence.

### 3.3 Fluids and the dura: neuraxial elasticity and compliance

Changes in neuraxial tissue compliance or elasticity lead to changes in kinematics and mechanical behavior, which can manifest anywhere else in the neuraxial system, as well as fascial dynamics or muscle control from the skull to the limb extremities. Anything affecting neuraxial tissue properties may impair the regulation of responses to mechanical loading, body motion, and fluid dynamics; local alterations transfer to remote areas of the neuraxis, impacting CSF dynamics and associated vasculature. Further, altered mechanical properties influence spinal joint mechanics, directly (via altered force vectors in the spinal canal or transferred via attachments of the neuraxis to the spine) or indirectly via effects on sensory feedback and motor control. Changing tissue compliance may cause or respond to hydrostatic effects of CSF pressure in the dural compartment or other changes in neuraxial tissues. Alterations of craniospinal compliance are associated with CSF pressure disorders—both high CSF pressure (idiopathic intracranial hypertension; IIH) and low CSF volume (spontaneous intracranial hypotension; SIH or CSF leaks). In IIH the spinal canal shows reduced compliance in MRI studies (55). Conversely, in SIH the intrathecal spinal canal shows increased compliance to infused fluid (73–76), and venous distension (a proposed diagnostic sign for SIH) is noted both intracranially and within the spinal canal (77, 78). Intracranially, MR elastography studies in SIH show increased stiffness in periventricular brain regions and decreased stiffness around the cerebellum and frontal lobe (79, 80).

Changes in dural compliance, fluid pressure and tissue architecture in the spinal canal affect kinematics of the whole neuraxial system, which may permanently change in response to a CSF leak and persist after sealing. Dural defects, like Type 1 and 2 spinal CSF leaks, will alter the distribution of mechanical stresses around and along the neuraxis due to the discontinuity of tissue fibers. Acute and chronic spinal CSF leaks have distinct symptom profiles; potentially due to tissue compliance and CSF dynamics changes over time in response to CSF pressure abnormalities (81). Despite this, calls to update the SIH diagnostic criteria to report both chronic and acute symptoms have not yet been implemented (82). “Scarring, fibrosis, and subsequent epidural compartmentalization” or “the formation of adhesion between the protruding arachnoid and the surrounding soft tissue” is hypothesized to reduce egress of CSF through the dural defect over time (81, 83). New membranous structures develop on the dura surrounding spinal CSF leak sites—“neo-membranes” or herniated arachnoid membranes—along with arachnoid blebs or pseudomeningoceles, which may persist beyond the apparent sealing of a CSF leak (83). Epidural scarring may occur after epidural blood patch (EBP) treatment, impacting dural mechanics, but the frequency with which this occurs is unknown (84). The short- and long-term biomechanical implications of these structural changes are not yet understood. No longitudinal studies have established whether compliance is restored to normal levels after successful CSF leak repair. Many questions remain unanswered: Do repaired dural defects regain the same mechanical properties as the original tissue? How restorative are different treatment approaches, and pre- peri- and post-operative protocols? How do physical therapies influence mechanical properties, optimize sliding between tissue interfaces, and guide restoration of fiber organization within the tissue through controlled loading during the tissue repair and remodeling phases?

### 3.4 Inflammation, congestion, and structural changes

Inflammatory processes impact neuraxial tissue plasticity, elasticity, and capacity to accommodate normal loading (30). Neural tissue inflammation is associated with psychiatric and physiological conditions, impaired motor control, proprioceptive deficits, venous congestion, and CSF pressure changes (85). As raised intra-abdominal pressure can increase vascular congestion in the spinal venous system, comorbid pathologies that may raise intra-abdominal pressure also deserve attention in attempts to prevent or manage conditions involving altered CNS fluid dynamics. This should include consideration of inflammatory, gastrointestinal and respiratory dysfunction, as well as compensatory patterns of trunk muscle function related to general skeletal instability.

Occult tethered cord is associated with changes in inflammatory infiltrates, tissue structure, vascular distention and reduced elasticity of the filum terminale (2, 62, 86). Pathological changes in filum elasticity may cause altered transfer of mechanical stresses to the spinal cord with resulting changes in neural function and subsequent symptoms. Altered mechanical loading of the filum (possibly due to the interaction of increased spinal range, connective tissue fragility and impaired proprioception) may result in vascular distention and altered venous drainage of the area, further impacting the filum mechanical properties. Venous congestion of the epidural veins may also arise as a result of extra-spinal venous compressions or lowered CSF pressure. Cauda equina syndrome may be attributed to epidural venous engorgement secondary to extra-spinal lesions and the resulting compression of lower spinal neural structures (87–89). Venous hypertension is associated with extravasation of inflammatory cells across the vascular epithelium in other tissues (90), and may be adding potential inflammatory effects to the compressive effects of epidural venous engorgement on the neuraxial tissues of any cause (including reduced CSF volume, venous compression syndromes) (90, 91). PET scans have detected inflammation in extradural tissues surrounding a spinal leak, potentially due to pro-inflammatory properties of extradural CSF or the dural tissue damage (92, 93). Further research is needed to understand intrinsic inflammatory interactions with dural perfusion, dural injury, EBP or surgical repair, and the implications of these inflammatory effects on dural healing times and mechanics with acute or chronic leaks, or persistence beyond leak sealing.

### 3.5 Compression, pressure, and entrapment mechanics

Due to the mechanical continuity of the neural tract, one or more sites of pathomechanics of the PNS have the potential to alter the neuraxial kinematics. Peripheral nerve entrapments and subluxations (common with EDS and Thoracic Outlet Syndrome, all underdiagnosed) (94), impact management of CNS pathology via the interplay of the PNS and CNS mechanics; CNS treatment may impact and be affected by peripheral nerve pathology, and vice versa. Targeted management of peripheral nerve pathologies may reduce pathomechanics that transmit aberrant forces to the neuraxis and attached structures; thus preventing or reducing kinematic factors that contribute to CNS pathologies (tethered cord, upper cervical instability, recurrent spinal dural tears).

EDS predisposes higher rates of venous compression syndromes involving peripheral vascular structures (70). Abnormal venous compression can contribute to intracranial or spinal venous congestion. Restricted outflow via the internal jugular vein (such as in Eagles Syndrome or Thoracic Outlet Syndrome) could contribute to cranial venous congestion (95). Hypertonic or hyperplastic suboccipital musculature related to upper cervical instability or altered skeletal kinematics or proprioception may also affect flow through the posterior cranial venous system Abdominal or pelvic venous congestion (due to compressions such as the nutcracker compression of the left renal vein) can result in backflow via collateral circulation into the spinal epidural veins (60) as noted in cadaver and animal studies, and clinical practice with EDS patients. Given the increased flow from pelvic veins to the spinal system in conditions of increased intra-abdominal pressure (60), sources of raised intra-abdominal pressure deserve more investigation as a potentially significant variable in CNS symptoms driven by increased CNS fluid pressures.

## 4 Treatment considerations

Multiple pathologies may contribute to CNS dysfunction with EDS, with a high likelihood of multiple coexisting, interacting pathologies in the one individual. Table 1 demonstrates the significant overlap of symptoms that pose a challenge for both clinical diagnostics and treatment planning. Ignoring the neuraxial interconnectedness and interacting pathomechanics between spinal motion, neuraxial motion, spinal muscle function, proprioception and CSF and vascular dynamics risks prescription of premature, delayed, ineffective or overly-invasive treatment options and unnecessary side effects due to unmanaged consequences of an intervention on other aspects of the system. A multidisciplinary focus that prioritizes neuraxial biomechanics and interactions throughout preventative care and treatment is crucial for responsible care to support long-term neuraxial regulation and neurological function.

**Table 1:** Symptoms of CNS disorders associated with EDS.

Common symptoms	UCI	SIH	IIH	Chiari
Headache	✓	✓	✓	✓
Neck pain	✓	✓		
Interscapular pain & stiffness	✓	✓		
Nausea/vomiting	✓	✓	✓	
Photophobia and phonophobia		✓	✓	
Orofacial numbness, weakness, or pain	✓	✓		
Vision changes *(blurred, tunnel, visual field defects, nystagmus, diplopia, and aura)*	✓	✓	✓	✓
Hearing changes *(hearing loss, tinnitus, and hyperacusis)*	✓	✓	✓	✓
Dysphonia	✓			
Dysphagia *(choking and trouble swallowing)*	✓	✓		
Disturbed balance	✓	✓		✓
Vertigo	✓			
Dizziness, disequilibrium	✓	✓	✓	✓
Altered taste		✓		
Pituitary dysfunction		✓		
Altered consciousness (stupor and coma)		✓		
Cognitive deficits, signs of dementia		✓	✓	✓
Movement dysfunction (ataxia, spasticity, and parkinsonism)	✓	✓		✓
Altered sleep architecture, sleep apnea	✓	✓		✓
Evidence of dysautonomia (eg, POTS)	✓	✓		✓
Signs of cranial nerve and brainstem compromise	✓	✓		✓
Sensory loss, paraesthesia	✓			

Overlapping symptom profiles for common CNS disorders associated with EDS and hypermobility, noting that some of the listed symptom categories taken from existing literature also overlap. Overlapping symptoms can complicate differential diagnosis of (1) Upper Cervical Instability (2, 149, 150), encompassing both CCI and AAI: (2) Spontaneous Intracranial Hypotension (82, 97, 135, 141), low CSF volume secondary to spinal CSF leak; (3) Idiopathic Intracranial Hypertension (71, 119), high CSF pressure or rebound pressure following sealing of CSF leak; or Chiari – (4) Chiari Syndrome (2, 71), cluster of symptoms that present with Chiari Malformation. CNS, central nervous system; EDS, Ehlers-Danlos syndromes; UCI, upper cervical instability; CCI, craniocervical instability; AAI, atlantoaxial instability; SIH, spontaneous intracranial hypotension; CSF, cerebrospinal fluid; IIH, idiopathic intracranial hypertension; Chiari, Chiari syndrome.

Where raised intracranial pressure is suspected, a comprehensive, multidisciplinary assessment may identify many contributing factors amenable to low risk intervention as first line treatment. Traditional treatments focus on relocating excess CSF via surgical shunting of CSF or surgical management of intracranial venous stenosis where weight loss and pharmacological management of intracranial pressure have failed. The positive effects of weight loss on IIH are likely moderated by changes in intra-abdominal pressure; so identifying contributing factors to increased intra-abdominal pressure may suggest other first line, non-invasive, low risk treatments. Contributing factors may include gastrointestinal, respiratory and trunk muscle dysfunction, all common in EDS and potentially manageable by dietary intervention, gastroenterology input, management of dysautonomia and physical therapies. Multidisciplinary treatment planning may consider a wider range of conservative treatment options prior to escalation to invasive or neurosurgical intervention. Other factors contributing to raised intracranial pressure include inflammation and disturbances of venous or lymphatic function outside the CNS, which result in impaired cranial venous and CSF drainage or venous backflow into the spinal epidural system. Such vascular and lymphatic insufficiencies may be modified by physical therapies to improve overall lymphatic function (96) or address musculoskeletal dysfunctions contributing to neurovascular compression, or to surgical intervention options in lower risk regions before considering higher risk vascular procedures.

Current spinal CSF leak treatment guidelines encourage early intervention to seal the leak in order to optimize outcomes (97, 98). But timely intervention is rare with non-iatrogenic SIH, and many factors influence the longevity of the dural closure and recovery of optimal CNS function and neuraxial kinematic control—especially with chronic leaks. Alterations in the biomechanical properties of the meninges and associated tissues may adversely impact both neurological function and the capacity of the dura to withstand loading associated with functional activity beyond the initial tissue healing phase. Treatments must aim to restore the dura’s functional capacity to withstand and dissipate normal forces associated with physiological movement and fluid pressure changes. Any regional change to the dura (elongation, tears, inflammation, adhesions) will influence force dissipation through the dural structures thereafter, potentially setting up pathological loading that could continue to adversely impact the dura, spine or remote areas of the neuraxis. Patient positioning immediately following EBP, therefore, should be optimizing travel of the blood bolus to the leak site, avoiding contracture or lengthening of areas of the dural tissue, and maintaining the capacity of the dura to move relative to associated tissue interfaces. Neutral spinal alignment supports the healing of the dura without stretching or folding the tissue. Body positioning relative to gravity determines the position of the neuraxis within the spinal canal; so “log rolling” motion could reduce adhesion formation by limiting sustained contact between the dura and adjacent spinal canal structures, while aiding distribution of the EBP bolus. As healing progresses, controlled loading and motion influences the fiber orientation and architecture of the dura to assist in re-establishing resilient tissue that maintains integrity under load and can induce positive effects on inflammation within neuraxial tissues (36, 99). Informed physical therapists can guide gradual progression of dural loading and recovery of spinal muscle control—reducing risk of further leaks short- and long-term—assisting recovery of general physical capacity while accounting for pathology cascades and comorbid conditions. Managing factors that add mechanical load to the dura could increase EBP success rates and reduce the likelihood of recurring leaks. Such factors include myodural load, any segmental instability of the spine (inducing nerve root stretch and vertebral encroachment on the dura), or tachycardia (amplifying the interacting effects of CSF volume and cord pulsatility).

In clinical presentations suggesting altered kinematics of the neuraxis and spine (upper cervical instability, tethered cord), comprehensive treatment planning considers all influences on kinematics to identify potential therapeutic targets. Physical therapy manages proprioceptive and motor control deficits and transmitted pathological forces due to peripheral nerve entrapments or myodural bridge overload (due to compensatory sub-occipital muscle activity), and venous distension due to various compression syndromes and aims to reduce mechanical loading of the neural system and musculoskeletal sources of nociceptive input. Injection based therapies, such as proliferative injections and hydrodissections, can be explored for their potential to improve kinematics by modifying soft tissue properties (100–103). Managing the inflammatory impacts of epidural venous congestion may reduce aberrant loading and mechanical property changes of the dura and filum terminale in tethered cord. Management of dysregulated hyaluronan or mast cell activation may also be indicated in the presence of atypical responses to anesthesia, imaging contrast, NSAIDs or exercise (25, 66). Managing tachycardia may also reduce the impact of altered cardiac synchronous spinal cord motion. Best practice integrates these measures into perioperative “prehab” planning when considering surgical spinal fusions or filum terminale excision. Comprehensive multidisciplinary treatment planning (managing neuraxial kinematics, spinal stability, fascial and fluid dynamics, proprioception, motor control, neuraxial loading, nerve entrapments, inflammation, tachycardia) has potential to reduce surgical risk and improve pre- and post-surgical outcomes, improve pain management, reduce complications or the need for repeated surgeries, and may reduce symptomatology and improve functional capacity enough to delay or avoid surgical intervention in some cases.

### 4.1 EDS, MCAS, POTS, and GI

The Ehlers-Danlos Syndromes (EDS) are a group of heritable disorders of connective tissue “characterized by joint hypermobility, skin hyperextensibility and tissue fragility” (104). Fourteen subtypes of EDS have been identified with genetic variants impacting the structure of fibers of the connective tissue, its volume, production, or overall architecture of the extracellular matrix (2, 105). Due to the ubiquity of connective tissue in body systems, EDS is associated with a broad range of symptomatology across multiple body systems, including gastrointestinal, urogynaecological, psychological and immunological manifestations (104, 106–108).

Mast Cell Activation Syndrome (MCAS) is increasingly recognized as a significant factor in EDS symptomatology, due to the diffuse actions of mast cell mediators throughout the body, inflammatory impacts, and trophic effects on connective tissue (107, 109–112). Mast cells are primarily located in tissues that interface with the external environment, and in the meninges, and mucosal and neurological tissue, with involvement in inflammation as well as immune responses, and gastrointestinal and neurological dysfunction (72). Dysautonomia has been reported to have twice the diagnosed prevalence of MCAS in EDS, which likely reflects healthcare access inequities and diagnostic delay rather than actual prevalence of the condition.

Autonomic dysfunction, most commonly presenting as Postural Orthostatic Tachycardia Syndrome (POTS) is commonly diagnosed in individuals with EDS (111, 113–115). With comorbid dysautonomia and mast cell activation syndrome, odds of EDS increases by 32 times, and dysautonomia mediates the association between hypermobility, chronic pain, and neurodivergence (116, 117). Reduced cerebral perfusion and compensatory tachycardia in the upright posture presents in many individuals with EDS, with increased compliance of vascular structures (allowing dependent blood pooling) and disordered autonomic neural regulation proposed as explanatory mechanisms (118). POTS may cause headache and pain in the cervical and thoracic region, attributed to reduced vascular perfusion of tissues in the cranial and upper trunk regions, and is also associated with SIH, UCI, and IIH (3, 111, 119–121).

A range of spinal and neurological manifestations are associated with EDS and hypermobility—peripherally: entrapments and subluxations of peripheral nerves (attributed to the lower resilience of the perineural connective tissue) (94) and small fiber neuropathy (122); centrally: symptomatic Chiari Malformation and segmental spinal instability (including at the craniocervical junction and upper cervical spine), Spinal CSF leaks, Tethered Cord Syndrome (TCS), spinal deformities (4, 5), migraine, Idiopathic Intracranial Hypertension (IIH), and early degenerative changes (2, 95). Impaired proprioception reduces kinematic control of skeletal articulations, and regulation of muscle forces during movement tasks (123, 124).

### 4.2 Spinal CSF leaks and spontaneous intracranial hypotension

Spontaneous Intracranial Hypotension (SIH) is low intracranial CSF volume in the absence of iatrogenic cause (post-puncture CSF leaks secondary to dural puncture or spinal surgery complications). Spontaneous CSF leaks may arise after whiplash or with no traumatic precipitant (125). Low CSF volume is the recommended descriptor with this condition, as normal CSF pressure on lumbar puncture is common and cannot rule out SIH or CSF leaks (82, 97, 98, 126). A significant proportion of patients presenting with SIH clinically have underlying connective tissue disorders (126–128), however studies are yet to confirm prevalence or incidence of SIH in EDS (129). Spontaneous CSF leaks are categorized based on the location of leakage: ventral dural tears (Type 1a); posterolateral dural tears (Type 1b); meningeal diverticula or dural ectasia (Type 2); and direct CSF-venous fistulae (Type 3) (82) and CSF-lymphatic fistulae recently described (130). Classically, SIH presents with orthostatic headache—headache that is worse when upright and better on lying down, although not consistently the case, especially with more chronic leaks, so cannot be relied on diagnostically. One study found that 24% had non-positional headache, and 16% had orthostatic symptoms present after up to 2 hours of upright posture (97, 131). The absence of orthostatic headache does not rule out SIH, as a subset of patients with confirmed CSF leak present without orthostatic headache; a wide range of other symptoms are common (Table 1) (97). SIH can severely reduce quality of life (132), with serious implications if untreated including superficial siderosis or spinal cord herniation (133), dementia (134), coma or death (135–137).

Negative results on whole-spine and brain MRI cannot rule out SIH, as false-negatives are common, and spinal MRI does not detect CSF-venous fistulae (representing an estimated 50% of leaks) contributing to significant diagnostic delays and misdiagnoses (97). Current radiology approaches seek to identify morphological changes in the brain with SIH, loss of CSF buoyancy, compensatory venous engorgement, or spinal epidural CSF collections. SIH can be misdiagnosed on imaging as Chiari malformation due to the presence of cerebellar tonsillar ectopia although certain radiographic measures may help differentiate the two conditions (71, 138). Studies have consistently demonstrated normal or high spinal CSF opening pressure in patients with confirmed leaks, making this an unreliable biomarker for a leak. Despite this, the diagnostic criteria still reference low opening spinal CSF pressure (40, 73, 139, 140). EBP treatment is recommended if SIH is suspected clinically, as a significant percentage of patients show positive treatment responses to EBP in absence of orthostatic headache, positive imaging, or low opening pressure (97, 141).

Treatment for SIH includes conservative measures of bed rest to encourage self healing, EBP or fibrin glue patch, surgical closure of dural tears, and embolization techniques for CVFs (30, 31, 82, 142). EBPs can be “targeted” directly to the leak site where it has been identified, or “non-targeted” (remotely in a location of convenience) but generally require larger volumes and repeated applications for spontaneous leaks in comparison to treatment of post dural puncture leaks (129). Shorter duration of pre-operative symptoms is the strongest predictor of outcomes for surgical repairs (81), supporting a call by clinical experts for earlier, more aggressive treatment to seal CSF leaks (98). Successful treatment can result in significant improvement in quality of life, however many patients experience only partial symptomatic recovery, recurring leaks or rebound intracranial hypertension, particularly if closure of the leak is delayed (81, 132, 140, 141, 143, 144).

### 4.3 Idiopathic intracranial hypertension

IIH presents with raised CSF pressure, reflected in opening pressures at spinal level, and papilloedema, with a range of associated symptoms. Management includes pharmacological interventions to reduce intracranial pressure, weight loss (which can indirectly reduce intracranial pressure), and physical redirection of CSF via insertion of shunts. While referred to as “idiopathic,” venous sinus stenosis is a common finding in patients with IIH, with stenting of the venous sinus proposed as treatment (145–147).

IIH is clinically associated with EDS, with the prevalence rate yet to be established (33, 79). An association has been reported between EDS, IIH, and extracranial cerebral venous outflow obstruction (34), with EDS patients having lower opening pressure, and co-occurring immunological, and neurological signs and symptoms not present in controls. IIH is a risk factor for development of CSF leaks and “rebound intracranial hypertension” can occur after sealing CSF leaks, necessitating careful management post-CSF leak repair to prevent rebound intracranial hypertension and recurrent CSF leaks (97, 126, 148).

### 4.4 Upper cervical instability

While emerging literature reports an association of UCI with EDS, the incidence of UCI in EDS remains unknown (3, 149, 150). UCI encompasses instability of the atlanto-occipital articulations (craniocervical instability, CCI) or the atlantoaxial articulations (atlantoaxial instability; AAI). While the term *CCI* is colloquially used to refer to both the instability of the atlanto-axial and atlanto-occipital articulations, the precise term *UCI* is used as the collective term here to prevent confusion. UCI has been associated with a range of symptoms of neuromusculoskeletal dysfunction as well as indications of “myelopathy, cranial nerve neuropathy, brainstem compression, vertebrobasilar artery compromise and compromised venous or cerebrospinal fluid outflow” and cerebellar dysfunction (149), as described in Table 1. The term “instability” refers to separate but related concepts of “mechanical instability” or laxity of passive joint restraints permitting excessive accessory joint motion and “functional instability” which is the subjective experience that a joint may sublux or “give way” due to insufficient neuromuscular control and differs from “hypermobility” which refers to increased range of physiological joint motion.

Inconsistencies remain in the diagnostic criteria and surgical management pathways for UCI in EDS, with a variety of radiological measures proposed as indicators of pathological joint motion (5, 150, 151). Clinical diagnosis is based on identifying indications of functional instability in the upper cervical articulations or mechanical instability of these joints together with neurological symptoms arising from the instability. Non-surgical management of UCI is currently guided only by clinical consensus guidelines, necessitating further research to clarify optimal approaches, treatment responsiveness and risk assessment for patient subgroups likely to benefit from non-surgical management (149).

### 4.5 Neurovascular entrapments

Recurring or widespread entrapment, impingement, compression, or subluxation of nerves or blood vessels may indicate underlying hypermobility or neuraxial dysfunction. Entrapment neuropathies and polyneuropathies commonly occur in the limbs (such as Carpal Tunnel or Cuboid Syndrome) and in the trunk (such as Median Arcuate Ligament Syndrome, Superior Mesenteric Artery Syndrome, May Thurner and Nutcracker compressions) (91, 94, 152). Compression of the proximal internal jugular vein in the space between the styloid process and C1 lateral mass has also been described in association with EDS and associated with intracranial hypertension (153).

Thoracic outlet syndrome (TOS) results in compression or impingement of arteries, veins or nerves passing through the thoracic outlet with movement of neck, arms and shoulders (154, 155); 90% of cases are neurogenic, and treatment options include physical therapy, decompression, or surgery. While certain sports, occupations, anatomical variants and shoulder girdle postures are known risk factors for TOS (154, 155), hypermobility is also a significant risk factor. One study found that 54% of hypermobile patients also had TOS symptoms (155), suggesting it as a potential target for diagnostic screening and both therapeutic and preventative intervention. In hypermobile athletes, electromyography shows myopathic or mixed neuropathic-myopathic patterns, which TOS researchers link to possible primary CNS impairment (154). History of entrapment and polyneuropathies should inform diagnostic assessment and treatment planning. Addressing underlying kinematic control and neuraxial capacity for muscle regulation may potentially reduce the frequency and severity of these neuropathies.

### 4.6 Tethered cord syndrome

A clinical presentation of progressive lower back and limb pain, bowel and bladder dysfunction and sensorimotor deficits in the lower limbs associated with a low lying conus medullaris on radiography has been well described as TCS. More recently, a similar symptomatic presentation without the low lying conus medullaris has been described in association with the Hypermobile subtype of EDS (hEDS) and termed Occult TCS (2, 35), with diagnosis based on the same clinical presentation without the radiological findings of TCS. Management of TCS by surgical release of the filum has been reported to result in similar improvements in patients diagnosed with occult TCS as compared to TCS with low lying conus (35, 156). The filum terminale of patients diagnosed as occult TCS have shown reduced elasticity, abnormalities of collagen fibrils and inflammatory cell invasion (35).

### 4.7 Chiari syndrome

Chiari malformation (CM) is typically characterized by caudal ptosis of the cerebellar tonsils through the foramen magnum (37). CM may be asymptomatic or associated with a range of symptoms (Chiari Syndrome), or may transition from asymptomatic to symptomatic in association with, whiplash, head/neck trauma, childbirth, dural puncture or spontaneously (38, 157).

An association exists between EDS and Chiari Syndrome, with ligamentous laxity contributing to functional changes at the craniocervical junction in CM (38, 157, 158). Altered craniocervical junction morphometrics detectable in upright imaging and reversible with cervical traction or return to supine posture have been noted in hypermobile CM patients that differ from non-hypermobile CM patients and may contribute to failure of surgical decompression alone to resolve symptoms (71, 158).

## 5 Biomechanical research directions

Connective tissue and biomechanical changes typify EDS, predisposing a range of CNS disorders, and these interactions remain underreported and overlooked in practice and research. Research and clinical education are needed into the interplay of neuraxial mechanics, tissue and fluid dynamics—with particular clusters of dysfunction, and changes over the lifespan—to identify potential risks and etiological factors relevant to the development, assessment, and treatment of different symptomatic presentations. Improved understanding of prevalence, progression, and mechanisms underlying common multimorbidity phenotypes could assist in earlier detection, intervention and preventative management of risk factors. Research suggests these CNS pathologies are associated with particular subtypes of EDS, most commonly the hypermobile type (hEDS) (2, 5, 159). While heterogeneity of presentation is noted in hEDS, a recent study identified three phenotypic clusters, one cluster showing a notably increased rate of neurological pathologies (4). As knowledge of the various genotypes and phenotypes of EDS develops, identifying specific risk factors, including kinematic and genetic variants, could inform timely diagnosis and preventative interventions in asymptomatic individuals with identified risk factors.

The craniocervical junction hosts a complex, multifaceted structural and functional interconnectedness of the neuraxial tissue and skeleton; presenting a prolific field for clinical and basic research. Dural mechanics, spinal kinematics, proprioception and CSF dynamics all have demonstrated patterns of influence via the myodural bridge and associated craniocervical structures, likely bidirectional patterns of influence. Complex chain reactions of pathology, stemming from any type of initial dysfunction, impact on this region. Initial pathology anywhere in the CNS that alters neuraxial biomechanics or sensorimotor function of the myodural bridge may lead to further alterations of joint kinematics, neuraxial loading and CSF dynamics, and adverse effects throughout the neuraxis.

Investigations of various biomechanisms *in context* may clarify predisposing factors and relative risk of deterioration, exacerbation, or developing additional pathology: mechanical properties of the neuraxis and spine in the context of CSF and vascular pressure disorders; CSF and vascular fluid dynamics in the context of spinal hypermobility and instability; spinal hypermobility and neuraxial loading in the context of increased tissue permeability (increasing osmolarity of porous tissues such as skin, gut, and blood brain barrier) and mast cell activation. A comprehensive understanding of these interactions guides treatment prioritization and planned sequencing of interventions where multiple pathologies impacting the CNS coexist (particularly with co-occurring or underlying EDS).

Research is needed to evaluate the role and efficacy of conservative therapies such as targeted motor control and proprioceptive training, and manual therapies to relieve sources of aberrant CNS tissue loading, vascular compression, neurovascular or lymphatic congestion, and to restore regulatory capacity subsequent to mechanical and other adverse effects. If mechanical risk factors can be identified earlier, physical therapies to manage or modify these risk factors may potentially delay, reduce, or avoid invasive surgical measures and the secondary complications and inherent risks of surgery in vulnerable patient populations.

Diagnostic delay is common and detrimental, due to the significant overlaps between symptom profiles of many CNS pathologies common in EDS. Combined with the absence of sensitive or specific diagnostic tests for these conditions, speed and accuracy of identifying contributing pathologies is lacking, and differential diagnosis is especially challenging. Research investigating the biomechanical characteristics of these disorders holds promise to add to existing diagnostic capabilities, with patterns of related mechanical dysfunction detectable in imaging studies. Further studies of CSF dynamics, cord motion and dural compliance parameters, may inform differential diagnosis and guide treatment prioritization by identifying mechanical patterns indicative of UCI, SIH, and other CNS mechanisms. Segmental spinal control and proprioception deficits contribute to aberrant mechanical loading of neurological structures, and may be further impaired by pathology of those structures. Research on spinal muscle activity patterns and proprioceptive function throughout the axial skeleton holds significant potential to illuminate contributing factors and potential therapeutic targets.

Beyond comparison with healthy controls, stratification or between-group analyses (EDS +/− UCI or EDS +/− SIH, for example) may examine the predisposing or causative role of hypermobility in altered cervical spinal cord motion, altered CSF dynamics or CNS pathology, and vice versa, further clarifying treatment priorities and preventative care targets. Between-groups comparisons of spinal muscle activity patterns and proprioceptive function could identify predisposing factors for the development or progression of CNS pathology, increased symptom burden and functional impairment. Investigation of the interactions between mechanical changes at different locations along the neuraxis and axial spine, proprioceptive function and motor control are needed to identify nuanced effects of neuraxial loading, risk factors, and precise therapeutic targets.

Altered CSF, vascular, and lymphatic fluid dynamics impact the mechanical properties of the dural and epidural tissue, with immediate effects on tissue compliance, elasticity, displacement, compression, and on the metabolic and immunological status of tissues. Altered fluid dynamics in the CNS, therefore, can have diffuse impacts on neuraxial tissues and connected structures to normal mechanical loading, and on proprioceptive functions, impacting progression or deterioration of CNS disorders. Investigation of neural biomechanics, proprioception and spinal muscle activity in the presence of altered fluid dynamics may clarify aetiological factors and therapeutic targets in management of disorders of CNS fluids (IIH, SIH, central venous engorgement). Further, longitudinal studies may indicate the progression of pathomechanisms and ascertain whether altered CNS fluid dynamics increases risk of structural changes such as tethered cord or upper cervical dysfunction long term.

## 6 Conclusion

Hypermobility has been flagged as a challenge across medical specialties, leading most fields to call for improved management approaches that account for the complexities of hypermobile patients. We have demonstrated that the biomechanical complexities introduced by hypermobility, connective tissue disorders, and related pathologies to neuraxial dynamics must be considered in diagnostics and treatment planning for neurological conditions. The more overt changes in neuraxial-myodural dynamics arising in the hypermobile physiology present the diverse medical disciplines with a unique opportunity to extend medical knowledge, and refine approaches to modulating neuraxial-myodural dynamics. With the rapid increase in the global burden of neurological diseases, there is an urgent need to enhance clinical education, diagnosis, and treatment of neuraxial dysfunction—so advances in hypermobile neuraxial care will potentially have far-reaching benefits across all facets of healthcare service and systems.
